# Production of Biodiesel from Nonedible *Parkia biglobosa* Oil under Acidic Condition

**DOI:** 10.1155/2023/3892348

**Published:** 2023-11-27

**Authors:** Rogers Kipkoech, Mohammed Takase

**Affiliations:** Department of Environmental Science, School of Biological Sciences, College of Agriculture and Natural Sciences, University of Cape Coast, Cape Coast, Ghana

## Abstract

In this study, biodiesel was produced from *Parkia biglobosa* oil via optimization of transesterification reaction conditions, (methanol to oil ratio, catalyst concentration, reaction temperature, and reaction time) under sulphuric acid catalyst (H_2_SO_4_). The oil was first extracted from *Parkia biglobosa* seeds using the Soxhlet extraction method. The physicochemical properties of the biodiesel were analysed and then compared to international standards. Subsequently, the oil was then used to produce biodiesel at optimized transesterification reaction conditions. The free fatty acid (FFA) content of the oil was 1.61% w/w, while the saponification value (mgKOH/g) was 191.65. The maximum yield (percentage weight) of the biodiesel produced was 93.4% at the maximum transesterification conditions of methanol-to-oil molar ratio of 6 : 1, sulphuric acid catalyst amount of 3 wt%, reaction temperature of 65°C, and reaction time of 1.5 h. The biodiesel produced was within the limits of international standards as per the specification by ASTM D6751 (American standard), EN 14241 (European standard), and Ghana Standard Authority. It was therefore recommended that biodiesel from *Parkia biglobosa* seed oil under acidic catalytic condition is a potential new substitute for petroleum diesel for commercialization purposes.

## 1. Introduction

Petroleum-based fuel performs a crucial function in the energy sector. Petroleum is used principally in the transport sector, specifically in motor engines to run vehicles and power plants. However, this resource is running out [1, 2]. Global warming is also becoming a concern as a result of anthropogenic activities such as burning of fossil fuels. However, there are significant efforts and interest to explore alternative sources of fuel which are renewable, sustainable and energy-friendly [3–5]. There is no doubt that renewable energy sources could provide the much-needed reprieve from the overutilisation of petroleum products.

Research has been extensively carried out on new, renewable energy sources in the quest of fulfilling the world's ravenous energy demand. The new substitute sources of energy include solar, wind, and biomass, among others. Biodiesel has become one of the most preferred forms of liquid fuel [6]. Biodiesel can, however, be compared in terms of characteristic to conventional petroleum diesel. It is renewable and produces minimal emissions after combustion compared to petroleum diesel. It could revolutionise into a sustainable fuel source [7, 8].

Biodiesel is produced through chemical processes such as transesterification with alcohol that are short-chain including methanol, butanol, and ethanol [9, 10].

The choice of an alcohol takes into consideration many factors including performance and cost-effectiveness. Ethanol and methanol are, however, primarily used as alcohols. Methanol is cheap and has desirable physical and chemical properties. On the other hand, ethanol is friendly to the environment since it is obtained from renewable sources such as corn, making the process entirely independent of petroleum-based alcohol [11, 12].

Kinematic viscosity of the oil from plants is supposed to be lowered to enhance its utilisation in diesel engines. Various methods have been developed to reduce oil viscosity. The methods are blending with diesel, transesterification, pyrolysis, and microemulsification [13, 14]. Blending and transesterification, however, remain the most popular methods. Meanwhile, among the two, transesterification remains a more superior and efficient method for reducing vegetable oil viscosity and yielding fuel with similar diesel properties [15, 16].

Transesterification entails the use of catalysts such as alkali, acids, or enzymes for biodiesel. The homogenous catalyst exists in the same phase (either gas or liquid) as the reactants. Typical homogenous cases include sulphuric acid, sodium hydroxide, and many others. Alkali catalysts are mostly preferred due to its practical and industrial feasibility. They are also less costly, unlike enzymes. Glycerol as the backbone of the reaction process is an essential component in soap-making and cosmetic industries [17, 18].

In this study, *Parkia biglobosa* oil has been used. In Ghana, biodiesel is being produced from edible oils such as rape seed, soybean oil, and castor. However, recent studies by Takase et al. [10] revealed that there are a lot of alternative nonedible high oil yielding feedstocks that can be considered as resources. One of the new and promising is *Parkia biglobosa* oil, which is gaining popularity in medicinal and pharmaceutical studies but very scanty in terms of a resource for biodiesel [15, 19, 20].

*Parkia biglobosa* is a member of the Fabaceae family which is widely grown in Ghana. The key regions that grow the plant are Northern Oti and Volta [20]. The active components of the plant have been exploited for medicinal and pharmaceutical purposes [19, 21]. Typically, the extract obtained from the leaves and seeds can be used to treat liver diseases [22]. Current studies have shown that the seeds from the plant contain a lot of oil [23]. According to studies by Olowokere et al. [23], the amount of oil per seed could be more than 11.4%. In addition, at Parkia industrial oil production in Volta Region, Ghana, the oil is seen as a by-product and goes to waste with little use. The purpose of this study was to investigate the biodiesel production from *Parkia biglobosa* seed oil using an efficient and economical sulphuric acid catalyst.

## 2. Materials and Method

### 2.1. Instrument

The instruments used in the experiments were Soxhlet extractor, 250 ml round bottom flask condenser, heating mantle, oven (DGH-9140A, YIHENG), furnace, GC mass spectrometer (GC-MS, 7890A Agilent Technology Inc., USA) and rotary evaporator (BÜCHI Rotavapor).

### 2.2. Reagents and Materials

Laboratory equipment and chemicals that were employed in the experiment included petroleum ether purchased from Ryht Aid Chemicals (Ho), electric grinder, 250 ml conical flask, condenser, magnetic stirrer, methanol of purity 98%, and 5% (w/w) H_2_SO_4_ obtained from the School of Biological Science Stores at the University of Cape Coast.

### 2.3. Experimental

#### 2.3.1. Sample Collection and Preparation

The collection of *Parkia biglobosa* seeds was done during the harvest season at Dambai in Oti Region. The samples of the seed were screened and thoroughly cleaned to ensure the elimination of dirt and seeds that have been destroyed for example by insects. Dehulling was carried out through the removal of the outer covering of the seed and washing, and then the seeds were sun-dried and subsequently grounded. The seeds were ground using an electric grinder into particle size and put into an airtight Teflon-lined container before extraction.

#### 2.3.2. Extraction of *Parkia biglobosa* and Determination of Percentage Yield

*Parkia biglobosa* oil was extracted using petroleum ether (40–60°C) by adopting the recommended method of Olowokere et al. [24]. The extraction of oil was done using a Soxhlet apparatus and petroleum ether as the extraction solvent. A ratio of 12 : 1 of solvent to seed was applied for extraction. 250 ml of the petroleum ether was poured into a round bottom flask while ground *Parkia biglobosa* seed weighing 25 g was kept in a filter. The filter containing the seeds was then carefully placed inside the thimble, fixed to the round bottom flask on top of the heating mantle, and connected to the condenser. The heating mantle was switched to heat the solvent [20].

The heated solvent in a conical flask began to boil and change to vapour and subsequently condense to liquid by a condenser. The extraction process was done in 5 h [23, 25]. The extracted *Parkia biglobosa* oil was then dried, recorded, and subjected to the degumming process by treating it with hot water to eliminate any hydrates, gums, phosphate, and other impurities. The content of the oil was determined using the method of Takase et al. [26].

#### 2.3.3. Composition and Characterization of the Feedstock

The content of the oil was analysed using Takase et al.'s [9] recommended method with little modification. Specifically, a thermal conductivity detector (TCD) was fitted to GC-MS and a high-polarity general purpose column was recommended for free fatty acids and phenols (15% FFAP, Chromosorb W, A/W 80/100, length of 2 m). Helium at a high purity of 99.9999% was used as the carrier gas. Column operation temperature in the range of 50°C–250°C and sample inlet temperatures of 200°C were used. The oven ramp temperature was initially maintained at 60°C for 5 min and thereafter increased to 220°C at 10°C/min for 10 min. Sample inlet pressure (carrier gas 20 ml/min) and total pressure with makeup (carrier gas and makeup 21 ml/min) were used. The temperature of the detector was 250°C. Analysis and characterization of *Parkia biglobosa* oil were done using the recommended method of Takase et al. [26].

### 2.4. Transesterification Reaction

The process of transesterification reaction of triglyceride to biodiesel was done using 20 g of the extracted *Parkia biglobosa* oil by initially filling into a 250 ml conical flask in a heating mantle connected with a condenser and magnetic stirrer. The weight of the oil was measured and recorded before heating to the standard temperature of 60°C. Methanol was then mixed with 4 wt% of H_2_SO_4_ and the solution was added to the oil. The methanolysis of triglyceride to biodiesel was done at varying methanol to oil ratios of 5 : 1, 5.5 : 1, 6 : 1, and 6.5 : 1. The time of the reaction was varied from 0.5 h to 2 h, the temperatures of the reaction from 55°C, 60°C, and 65°C to 70°C, and the amount of catalysts from 1% to 4% (weight of the oil). The yield of biodiesel was determined using the recommended method of Takase et al. [9].

### 2.5. Determination of Biodiesel Properties

The samples of biodiesel were analysed using the ASTM D6584 method [27] with little modification. Thus, a thermal conductivity detector (TCD) was fitted to GC-MS and a high-polarity general purpose column was recommended for free fatty acids and phenols (15% FFAP, Chromosorb W, A/W 80/100, length of 2 m). Helium at high purity of 99.9999% was used as the carrier gas. The column operation temperature ranging from 50°C to 250°C and sample inlet temperature of 200°C were used. The oven ramp temperature was initially maintained at 60°C for 5 min and thereafter raised to 220°C at 10 ^o^C/min for 10 min. Sample inlet pressure (carrier gas 20 ml/min) and total pressure with makeup (carrier gas and makeup 21 ml/min) were used. The temperature of the detector was 250°C.

The physicochemical properties of the biodiesel were analysed using the ASTM 6751 method and European standard (14214) as in Takase et al.'s study [26]. The properties analysed include density at 20°C, kinematic viscosity, acid value, water/moisture content, flash point, cold flow properties, cetane number, ash content, sulphur content, free glycerine, total glycerine, oxidative stability at 110^o^C/h, cold filter plugging point (°C), iodine value g/100, and copper strip corrosion (50°C; 3 h) [20, 28].

## 3. Results and Discussion

### 3.1. Physicochemical Characterization of *Parkia biglobosa* Oil

From the results of the study, the stearic acid for the analysed *Parkia biglobosa* oil was 4.30% while the reported *Parkia biglobosa* oil was 5.3%. The variation in the percentage of stearic acid in this study was slightly low. Similarly, linoleic acid for the *Parkia biglobosa* oil that was analysed and the one reported was 65.42% and 63.5%, respectively. The fatty acid composition of the obtained oil was similar to the study by Augustine et al. [29] as shown in Table 1.

The difference in palmitic acid in both oils agrees with the research by State [30]. In his research, State [30] alluded that the differences in the chemical composition of similar oil were due to differences in the locations where the oil was obtained. Some of the key factors such as soil type and climatic conditions were proven to affect the properties of the oil. Furthermore, these findings are comparable with those in the research by Lin and Lin [31], which found out that C16:0, C18:2, and C18:1 form the three main compounds. Furthermore, it was revealed that individual kinetics for the three acids mentioned are useful, especially in situations where the composition of FFA is higher, it will result in the biodiesel that has highest saturated fatty acid and hence huge thermal and oxidative stability [32]. When compared with Augustine et al.'s study [29], arachidic acid (5.21%) in this study was relatively high.

### 3.2. Properties of *Parkia biglobosa* Oil

*Parkia biglobosa* oil was analysed for physicochemical properties which include density, kinematic viscosity, water content, average molecular weight, and free fatty acid (Table 2).

Methods used for analysis were as outlined by the Association of Official Analytical Chemists [20, 28, 33] and American Standard for Testing Material (ASTM). Based on the data provided in Table 2, *Parkia biglobosa* oil has a high density of 916.2 kg/m^3^ and kinematic viscosity (35.70 mm^2^/s) at 40°C.

Denser oil produces a higher amount of energy [34, 35]. Biodiesel's relative density is important in mass to volume conversion and determines the flow and viscosity of properties. It is also helpful in the determination of the homogeneity of biodiesel. The saponification value as per the analysis of the *Parkia biglobosa* oil was 191.65 (mgKOH/g). The saponification value from the analysis still falls within the limit outlined by ASTM 4052-96. *Parkia biglobosa* oil contained high FFA of 1.61% and high water content of 1.93 (%) w/w.

Fatty acid and moisture contents are crucial parameters in determining whether oil is recommended for the transesterification process. Feedstocks containing high free fatty acid (>0.5%) cannot be easily converted by base catalyzed transesterification due to the concurrent formation of soap. That challenge was avoided through transesterification using the sulphuric acid catalyst [36, 37]. From the results in Tables 1 and 2, it was clear that *Parkia biglobosa* oil was ideal for biodiesel production.

### 3.3. Optimization of Reaction Parameters

#### 3.3.1. Methanol to Oil Ratio

**Figure 1:**
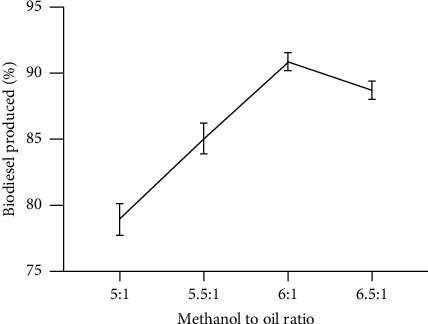
Influence of methanol-to-oil ratio on the production of biodiesel (reaction temperature 60°C, catalyst concentration 1 wt%, and reaction time 1 h); mean with standard deviation values were 78.93 ± 1.18, 85.07 ± 1.16, 90.90 ± 0.7, and 88.70 ± 0.7. Source: lab analysis (2021).

Figure 1 shows the influence of methanol on *Parkia biglobosa* oil. As shown in Figure 1, there was a gradual increase in biodiesel yield from 78.9% to 90.7% as the ratio of methanol to oil was adjusted upwards from 5 : 1 to 6 : 1, respectively. Optimization was carried out to determine the optimum biodiesel yield (90.7%) which was attained at the ratio of 6 : 1. There was a decrease in the biodiesel yield from 90.7% to 88.7% at the ratio of methanol to oil of 6.5 : 1. This was possibly due to an increasing molar ratio which leads to low segregation of glycerol and biodiesel due to high solubility [38]. According to Takase et al. [39], the alcohol to oil molar ratio is one of the crucial factors that affect the efficient methanolysis of oil to biodiesel and the cost of production. The results of this study are similar to Kipkoech [20] studies on the Production of Biodiesel from *Parkia biglobosa* oil using the heterogeneous bifunctional clay catalyst. In Kipkoech studies, the optimum methanol to oil ratio was 6 : 1 with a yield of 90.7%. The transesterification stoichiometric ratio of alcohol to oil is 3 : 1. Greater ratios are needed since the reaction is reversible [27].

Shifting the reaction to the completion side means the ratio is supposed to be higher than the stoichiometric ratios. Breaking glycerine-fatty acid bonds in the transesterification reaction of glyceride to form biodiesel requires extra alcohol [15]. Thus, a greater ratio of alcohol to oil leads to converting the large amount of alkyl ester within a short period [40]. This is supported by studies by Takase et al. [27] and Jabeen et al. [5]. Oil from nonedibles including pongamia and neem needs much alcohol to yield the maximum amount of biodiesel. This is due to the high viscosity of the nonedible oil as compared to edible ones [40, 41]. This assertion is in line with Munir et al. [35], who indicated that oil with a relatively high amount of viscosity needs to be reduced by esterification before transesterification.

#### 3.3.2. Reaction Temperature

**Figure 2:**
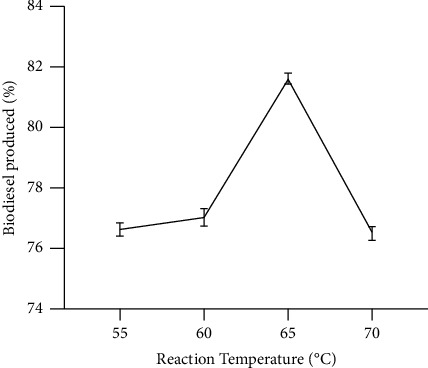
Influence of reaction temperature on the production of biodiesel (molar ratio of methanol to oil 6 : 1, catalyst concentration 1 wt%, and reaction rime 1 h); mean with standard deviation values were 76.63 ± 0.38, 77.03 ± 0.49, 81.63 ± 0.31, and 76.5 ± 0.4. Source: lab analysis (2021).

The influence of the temperature of the reaction on the yield of biodiesel was examined (Figure 2). The synthesis of biodiesel by methanolysis of *Parkia biglobosa* seed oil was achieved through variations of the reaction temperatures (55°C, 60°C, 65°C, and 70°C). As the reaction temperature was adjusted upward from 55°C to 60°C, it led to a slight rise in the yield of biodiesel from 76.6% to 77.0%, respectively. However, a sharp rise in biodiesel yield from 77.0% to 81.6% occurred when reaction temperature increased from 60°C to 65°C and decreased from 70°C to 76.5%. Therefore, the optimized reaction temperature for the transesterification of *Parkia biglobosa* oil was 65°C. The yields at reaction temperatures below and above 65°C were low. At 55°C and 60°C, the reaction was not complete. At reaction temperature above 70°C, the methanol started to burn entirely as some vaporize and formed bubbles. Since methanol undergoes melting at a temperature between 60°C and 65°C, the reaction interface is inhibited. These findings are in line with those of Borugadda and Goud [14] which noted similar observation on the vaporization of methanol at 70°C and the formation of bubbles [6]. Furthermore, it was noted that at 70°C, the reaction was still not complete. This observation could be because of a slight increase in the viscosity of the oil which results in oil and the catalyst mixing poorly [20, 28, 42].

#### 3.3.3. Effects of Catalyst Concentration

**Figure 3:**
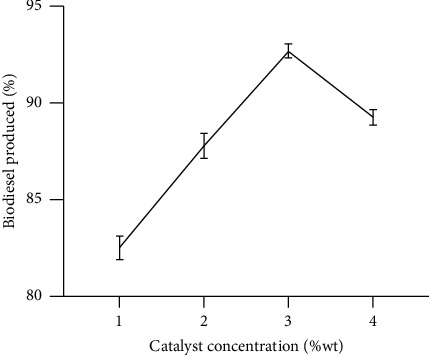
Influence of catalyst concentration on the production of biodiesel (molar ratio of methanol to oil 6 : 1, reaction temperature 65°C, and reaction time 1 h); mean with standard deviation values were 82.53 ± 1.07, 87.80 ± 1.13, 92.70 ± 0.61, and 89.27 ± 0.67. Source: lab analysis (2021).

Figure 3 indicates the influence of catalyst concentration on the yield of biodiesel. 1, 2, 3, and 4 wt% (weight) of the sulphuric acid concentration (with reference to the oil's weight) was used (Figure 3). Initially, there was a sharp increase in biodiesel from 81.3% to 93.4% as the amount of catalyst was raised from 1 wt% to 3 wt%. The optimum yield of 93.4% was attained by using 3 wt% of the catalyst. A sharp decline in the biodiesel yield (88.5%) was noted as the catalyst concentration increased to 4 wt%. Studies by Arumugam and Sankaranarayanan [43] showed similar trends. They highlighted that the rates of reaction in acid-catalyzed transesterification reaction can lead to the attainment of a higher yield of biodiesel by using a higher amount of catalyst. Increasing the concentration of catalyst results in more active sites for the methanolysis of available triglyceride. Additional upward adjustment in catalyst concentrations (above 4 wt%) resulted in a slight reduction in the yield [20, 44]. This could be as a result of the formation of soap (Figure 3).

Takase and Kipkoech [15] studied the methanolysis of grease using methanol with varied sulphuric acid concentrations (1, 3, and 4 wt%). The study explored the rate of enhancement of the rise in the level of catalyst concentration and the consequent yield of biodiesel. The optimum yield obtained in their work was 95.0%.

#### 3.3.4. Effects of Reaction Time

**Figure 4:**
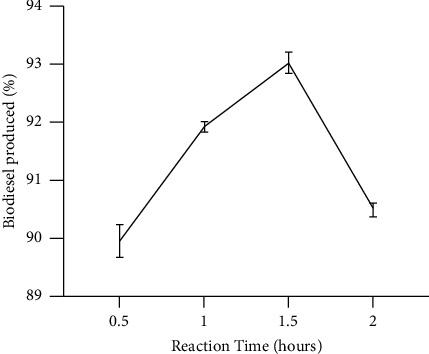
Influence of reaction time on the Production of Biodiesel (molar ratio of methanol to oil 6 : 1, reaction temperature 65°C, and catalyst concentration 3 wt%); mean with standard deviation values were 89.97 ± 0.49, 91.93 ± 0.15, 93.03 ± 0.32, and 90.50 ± 0.2. Source: lab analysis (2021).

Transesterification reactions depend on time. The influence of reaction time on the yield of biodiesel is shown in Figure 4. At 0.5 h, the yield of biodiesel was 90.0% (Figure 4). The reaction was, however, slow and this could be attributed to insufficient stirring which could not enhance adequate mixing and dispersion of methanol and oil [45]. In contrast, between 0.5 h and 1 h, a constant rise in the amount of biodiesel was obtained (90.0% to 91.9%). It was then preceded by a rise in the yield of biodiesel (93.0%) at 1.5 h of the reaction time and this was an optimum yield.

As the time was prolonged from 1.5 h to 2 h, the amount of biodiesel decreased from 93.0% to 90.5%. The reduction in the quantity of biodiesel obtained is as a result from the occurrence of a reversible reaction. The findings are similar to Takase et al.'s [9] findings on the influence of time on acid-catalyzed transesterification which resulted in a high amount of biodiesel produced (maximum) as the time of the reaction was increased. In the beginning, the slow rate of reaction could be due to poor mixing of methanol, oil, and the catalyst interface. Nevertheless, the optimum production of biodiesel was reached in less than 1.5 h. This shows the optimum amount of biodiesel from *Parkia biglobosa* seed oil was attained after a considerable time while using the H_2_SO_4_ catalyst. When compared with other oils which can produce optimum yields of biodiesel within a short time, the yield in this case is relatively low [9]. Further, the rise in the reaction time did not lead to a rise in biodiesel yield [15, 20, 46]. The reduction in biodiesel produced is because of further upward adjustment in reaction time which may have been caused by the backward reaction [9, 38]. This same trend was observed by Kipkoech [20] in the transesterification of *Parkia biglobosa* oil to biodiesel using sulphur acid as the catalyst under the same reaction condition (molar ratio of methanol to oil 6 : 1, reaction temperature 65°C, and catalyst concentration 3 wt%).

#### 3.3.5. Characterization of *Parkia biglobosa* Seed Oil and Biodiesel

**Figure 5:**
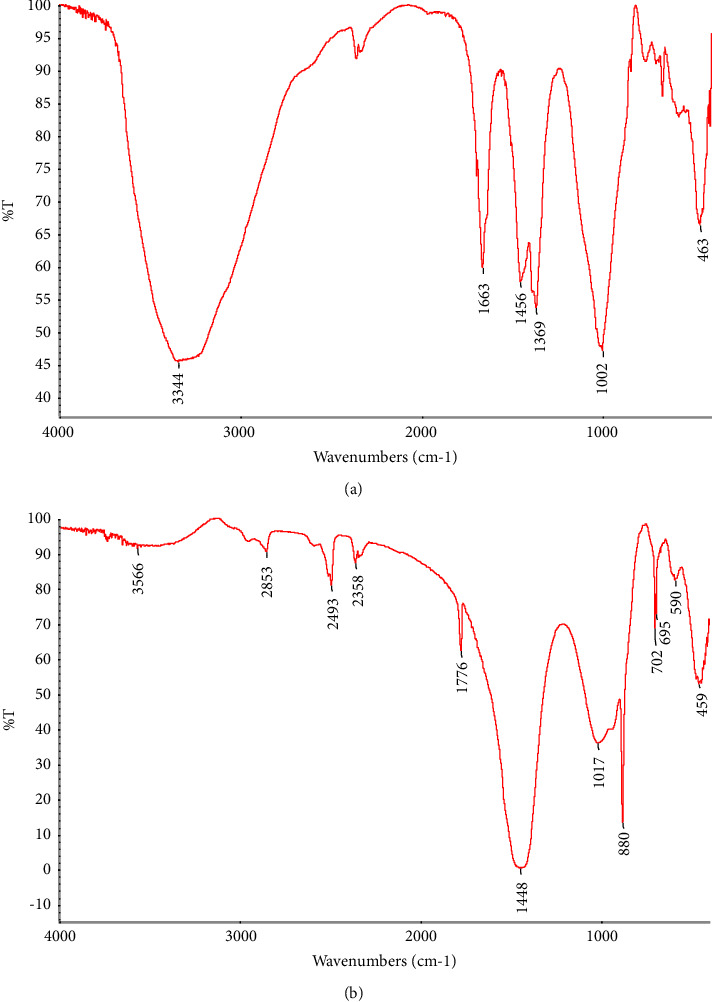
(a) FT-IR spectrum of *Parkia biglobosa* seed oil. (b) FT-IR spectrum of *Parkia biglobosa* biodiesel.

The FT-IR patterns of *Parkia biglobosa* oil and its biodiesel are indicated in Figures 5(a) and 5(b) respectively. There appeared a broad band at 3344 cm^−1^ of the oil. However, in the biodiesel, the broad band was weak and appeared at 3566 cm^−1^ and this could be attributed to the stretching vibration of the biodiesel. The bands at 1663 cm^−1^ for the oil and 1446 cm^−1^ for the biodiesel were ascribed to the stretching vibration of –CH_2_. The bands at 1369 cm^−1^ and 1017 cm^−1^ for oil and biodiesel were ascribed to vibration in the oil and biodiesel and the bands at 1002 cm^−1^ of oil and 860 cm^−1^ of the biodiesel were due to –C=O stretching vibration. The peak at 702 cm^−1^ of the biodiesel was due to shear-type vibration. The bands between 463 and 1002 cm^−1^ for the oil and between 459 and 590 cm^−1^ for biodiesel were due to the vibration of C-O-C group. The *Parkia biglobosa* oil band stretching from 3200 to 1700 cm^−1^ regions as well as *Parkia biglobosa* biodiesel band stretching from 3000 to 1400 cm^−1^ regions are endothermic peaks possibly as a result of very weak bonding vibration of the C-O-C group [20, 44]. The FT-IR outcome of the current studies is in line with the studies by Munir et al. [44] on the optimization of novel *Lepidium perfoliatum* Linn biodiesel using the zirconium-modified montmorillonite clay catalyst.

#### 3.3.6. Properties and Application Feasibility of *Parkia biglobosa* Biodiesel

Physical and chemical properties of the biodiesel prepared from *Parkia biglobosa* seed oil using sulphuric acid as a catalyst are within the acceptable standards (Table 3). The international standards used were ASTM D6751 and EN 14214 and the Ghana Standard Authority which describe biodiesel testing parameters and limits. Density and kinematic viscosity are crucial properties because they affect the performance of the engine. Fuel with higher density has greater mass and a more viscous one has a low flow rate which influences the engine output. Kinematic viscosity allows the fuel to flow freely into the injection during the combustion process. In the study, the viscosity of the oil was greatly reduced by 88.5% (from 35.70 to 4.1 mm^2^/s) in the biodiesel obtained. The oil density was 916.2 kg/m^3^ and that of the biodiesel was 868 kg/m^3^ [20]. The properties of the biodiesel in this case are consistent with Munir et al. [18] on their work on practical approach for the synthesis of biodiesel via nonedible seed oils using the trimetallic-based montmorillonite nano-catalyst.

The iodine value represents the degree of unsaturation of biodiesel. There was a significantly high level of iodine value (137 g/100) in the biodiesel which was above the limit for the acceptable standard (ASTM Std. D6751 and EN 14214). Iodine value is direct indication of the level of unsaturation in oils. The double bonds are a major source of unsaturation. The more the C=C bonds present in the fatty acids, the greater the value of the iodine number and the more the unsaturation. According to Munir et al. [18], producers of engine for a long time have been arguing that a high value of iodine possibly causes polymerization and formation of deposits on the engine nozzles, piston rings, and piston ring grooves during heating. There was 0.093% (w/w) water content in the oil which was not within the ASTM Std. D6751 of 0.05 as maximum. Water content in the biodiesel was 0.07 mg/kg which was slightly higher than the ASTM Std. D6751 and EN 14214 standard as well as the Ghana Standard Authority of 0.05 mg/kg maximum. The higher water content is due to biodiesel having higher affinity to moisture content and also having water retaining capacity after the transesterification process as compared to conventional diesel fuel. The other properties including cetane number, kinematic viscosity, and flash point were within international standards [20, 44].

#### 3.3.7. Summary of Optimum Reaction Parameters for the Production of Biodiesel from *Parkia biglobosa* Oil Using Sulphuric Acid Catalyst

Table 4 shows the optimum reaction conditions for the production of biodiesel from *Parkia biglobosa* seed oil through transesterification using sulphuric acid as a catalyst. According to the results presented in Table 4, the methanol-to-oil molar ratio was 6 : 1, while the reaction temperature was 65°C, and the concentration of the catalyst was 3 wt%, while the reaction time was 1.5 h. This experiment resulted in the highest amount of biodiesel yield of 93.4%.

## 4. Conclusion

*Parkia biglobosa* oil was obtained by subjecting the *Parkia biglobosa* seed to the Soxhlet extraction. The percentage yield of the oil was 16.5%. Properties such as fatty acid compositions, water content, saponification value, and many others of the oil as required were within acceptable limits for producing biodiesel. From the study, the following can be concluded: the highest biodiesel yield from *Parkia biglobosa* oil through transesterification with the sulphuric acid catalyst was achieved at a ratio of methanol to oil of 6 : 1, temperature of the reaction 65°C, and catalyst concentration of 3 wt% in 1.5 h. The optimum biodiesel yield was 93.4% [20]. The key properties of the obtained biodiesel were within the United States of American standard (ASTM D6751), European standard (EN14241), and Ghana Standard Authority. On the basis of the yields and the properties of the biodiesel as comparable to international standards complement with the abundance of *Parkia biglobosa* oil and at *Parkia* industrial production as a by-product in Ghana, the oil could be seen as a potential nonedible feedstock for biodiesel, particularly in Ghana. From Table 3, the properties of biodiesel from *Parkia biglobosa* indicate that the carbon footprint of transportation and other industries could be decreased using biodiesel made from *Parkia biglobosa*, which emits fewer greenhouse gases than conventional fossil fuels. Additionally, biodiesel produced from *Parkia biglobosa* oil can help diversify energy sources, lessen reliance on a single energy source, and improve energy security. Also, biodiesel production from *Parkia biglobosa* would offer some economic opportunities for local communities in Africa, including farmers who cultivate the *Parkia biglobosa* plant and those who extract the oil for biodiesel production. The study is also expected to contribute to enhancement and reduction of greenhouse gas emissions, regional development, and social structure, especially to developing countries such as Ghana. The study is also expected to bring a balance between agriculture, economic development, and the environment. The biodiesel produced will help improve the lubrication properties of the diesel-fuel blend which will subsequently help reduce long-term engine wear in diesel engines since biodiesel is known to be a good lubricant.

It is therefore recommended that biodiesel from *Parkia biglobosa* biodiesel should be tested in diesel engines to assess the engine performance and emission characteristics and the blends of *Parkia biglobosa* biodiesel with conventional diesel should be studied in order to assess the performance of the blends and also to improve on the flash point of the biodiesel (https://www.researchsquare.com/article/rs-2552401/v1).

## Figures and Tables

**Table 1:** Fatty acid composition of *Parkia biglobosa* oil [20].

Fatty acid composition	Analysed value (%)	Reported by [29] (%)
Palmitic acid (16:00)	15.08	5.0
Stearic acid (C18:0)	4.30	5.3
Oleic acid (C18:1)	7.90	12.5
Linoleic acid (C18:2)	65.42	63.5
Arachidic acid (C20:0)	5.21	—

**Table 2:** Properties of *Parkia biglobosa* oil used as feedstock for biodiesel production [20, 28].

Property	Testing procedure	ASTM Std.	Ghana Std. Authority	Determined value
Density at 20°C (Kg/m^3^)	ASTMD4052-96	860–890	870–900	916.2
Saponification value (mgKOH/g)	ASTMD4052-96	191–202	—	191.65
Kinematic viscosity at 40°C, mm^2^/s	ASTMD 445-06	1.9–6.0	1.5–6.5	35.70
Water content (%) w/w	AOAC 984.20-90	0.05 max	0.05 max	1.93
Free fatty acid content (%) w/w	AOAC 940.28	—	—	1.61

**Table 3:** Properties of *Parkia biglobosa* biodiesel compared to standards of Europe and United States and Ghana Standard Authority.

Property	Method	ASTM Std. D6751	EN 14214	Ghana Std. Authority	Determined value
Cetane number	ASTM D6890	≥47	≥51	47 min	51
Kinematic viscosity (mm^2^/s; 40°C)	ASTM D445	1.9–6.0	3.5–5.0	1.5–6.5	4.1
Oxidative stability (h)	EN 14112	≥3	≥6	—	3.1
Cloud point (°C)	GB/T510	−15 to 16	—	—	−1
Pour point (°C)	GB/T3535	−3 to 12	—	+15 max	−1
Flash point (°C)	ASTM D93	≥93	≥120	66 min	155
Sulphur content (%, w/w)	ASTM D4294	≤0.05	0.020	0.05 max	0.004
Ash content (%, w/w)	GB/T508	≤0.02	≤0.02	0.1 max	0.019
Acid value (KOH mg/kg)	ASTM D664-01	≤0.5	≤0.5	1.0 max	0.456
Water (mg/kg)	D6304	≤0.05	≤0.05	0.05 max	0.07
Density (20°C)	SH/T0248	—	860–900	—	868
Iodine value (g/100)	EN 14111	≤120	≤120	—	137
Copper strip corrosion (50°C, 3 h)	ASTM D 130-94	≤No.3a	≤No.1a	—	1a
Free glycerine (%, w/w)	EN 1405	≤0.02	≤0.020	—	0.002
Total glycerine (%, w/w)	SH/T0796	0.24	≤0.25	—	0.16

Source: lab analysis (2021).

**Table 4:** Optimum reaction conditions for the production of the biodiesel.

Factors	Optimum conditions
Methanol-to-oil molar ratio	6 : 1
Reaction temperature (°C)	65
Catalyst concentration (wt%)	3
Reaction time (h)	1.5
Biodiesel yield (%)	93.4

## Data Availability

The data used in this research are available within the article.
